# The Association of Hypertension with Increased Mortality Rate During the COVID-19 Pandemic: An Update with Meta-analysis

**DOI:** 10.1007/s44197-023-00130-3

**Published:** 2023-06-15

**Authors:** Ahmad R. Al-Qudimat, Ayisha Ameen, Doaa M. Sabir, Heba Alkharraz, Mai Elaarag, Aisha Althani, Kalpana Singh, Wassim M. Alhimoney, Raed M. Al-Zoubi, Omar M. Aboumarzouk

**Affiliations:** 1 Surgical Research Section, Department of Surgery, Hamad Medical Corporation, Doha, Qatar; 2 Department of Public Health, QU-Health, College of Health Sciences, Qatar University, Doha, Qatar; 3 Department of Biomedical Sciences, QU-Health, College of Health Sciences, Qatar University, 2713 Doha, Qatar; 4 Department of Chemistry, Jordan University of Science and Technology, P.O.Box 3030, Irbid, 22110 Jordan; 5 College of Medicine, Qatar University, Doha, Qatar; 6 School of Medicine, Dentistry and Nursing, The University of Glasgow, Glasgow, UK; 7 Nursing Research Department, Nursing Corporate, Hamad Medical Corporation, Doha, Qatar

**Keywords:** HTN, COVID-19, Mortality, Prevalence, Pandemic, Chronic disease

## Abstract

**Background and Aim:**

The impact of multiple risk factors on COVID-19 mortality has been previously reported in multiple systematic reviews and meta-analyses. The aim of this review is to provide a comprehensive update on the association between hypertension (HTN) and mortality in patients with COVID-19.

**Methods:**

A systematic review and meta-analysis were performed and followed the Preferred Reporting Items for Systematic Reviews (PRISMA) guidelines. A search was achieved using PubMed, Scopus, and Cochrane Databases for research publications on hypertension, COVID-19, and mortality published between December 2019 and August 2022.

**Results:**

A total of 23 observational studies involving 611,522 patients from 5 countries (China, Korea, the UK, Australia, and the USA) were included in our study. The confirmed number of COVID-19 with HTN cases in each study ranged from 5 to 9964. The mortality ranged from 0.17% to 31% in different studies. Pooled results show that the mortality rate of COVID-19 among the included studies ranges from a minimum of 0.39 (95% CI 0.13–1.12) to a maximum of 5.74 (95% CI 3.77–8.74). Out of the 611,522 patients, 3119 died which resulted in an overall mortality prevalence of 0.5%. Subgroup analyses indicated that patients with COVID-19 who have hypertension and male patients had slightly less risk of mortality than female patients [the percentage of men > 50%; OR 1.33: 95% CI (1.01, 1.76); the percentage of men ≤ 50%: OR 2.26; and 95% CI (1.15, 4.48)]. Meta-regression analysis results also showed a statistically significant association between hypertension and COVID-19 mortality.

**Conclusion:**

This systematic review and meta-analysis suggest that hypertension may not be the only risk factor associated with the increased mortality rate during the COVID-19 pandemic. In addition, a combination of other comorbidities and old age appears to increase the risk of mortality from COVID-19.

**Graphical Abstract:**

**Figure:**
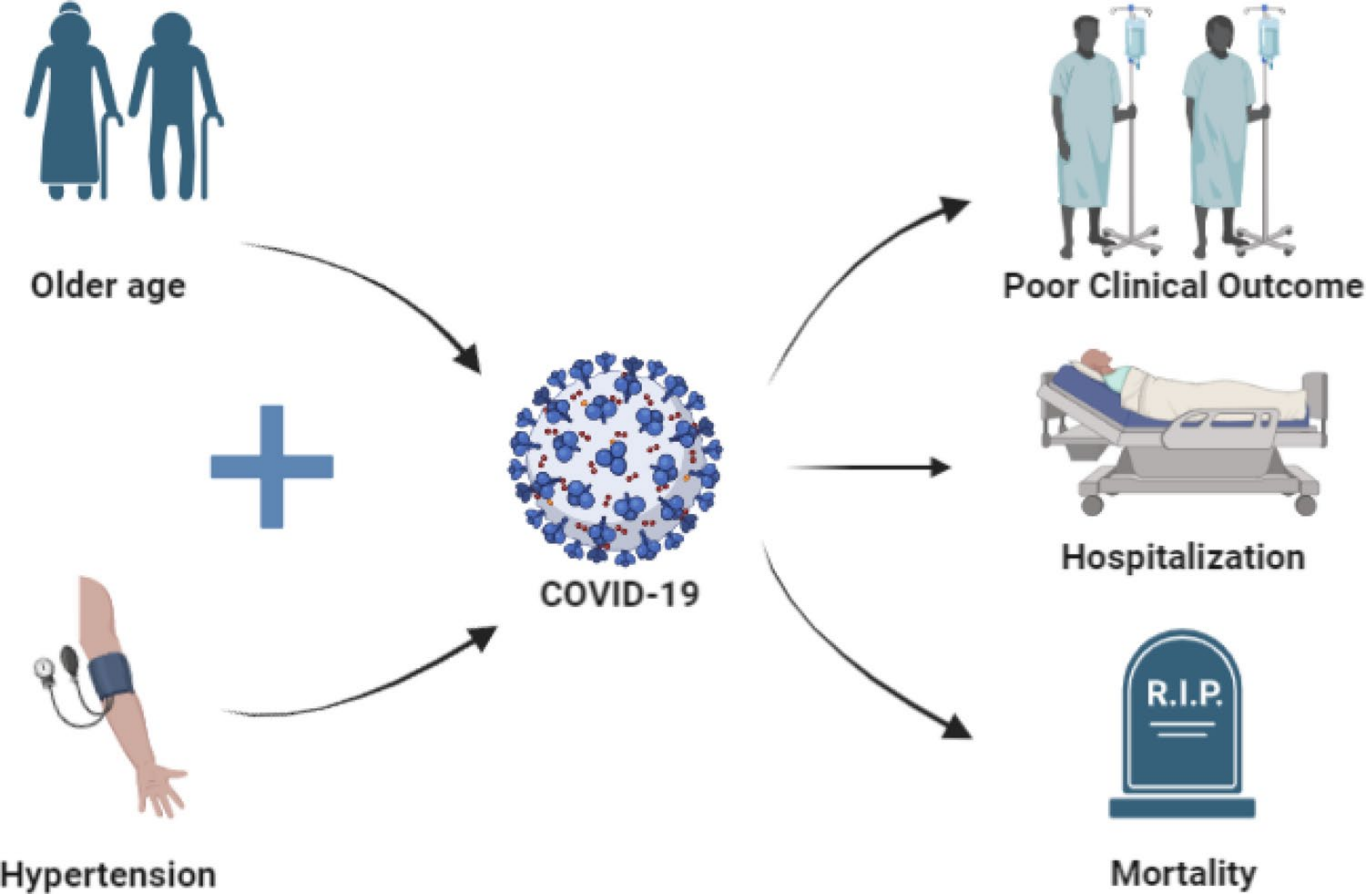
The impact of hypertension on mortality rate among COVID-19 patients

## Introduction

Since November 2019, the world has been pushed into a very challenging era due to COVID-19 pandemic [1]. The disease course of people infected with COVID-19 varies from asymptomatic to mild, moderate or severe symptoms, sometimes requiring admission to intensive care units. Some patients may develop severe symptoms led to complications such as pulmonary failure, cytokine storm, and multi-systems failure, eventually contributing to their death. Many conducted studies reveal that there are several factors predisposing COVID-19 patients to the stage of severe infection and death. Among such various risk factors, are older age, male vs. female, and those with various comorbidities [2]. Through the literature, it is evident that arterial hypertension disease is a risk factor that leads to a high risk of morbidity and mortality in patients with COVID-19 [3].

Hypertensive patients are at a higher risk of acquiring COVID-19 infection and experiencing various complications. However, the exact cause of this relationship is still unclear. Several possible reasons have been suggested, including the presence of cardiac damage due to long-standing hypertension, the potential interaction between COVID-19 and widely used medications for hypertension treatment, and the higher incidence of hypertension in the elderly population.

As several studies have been reported, older people are more prone to acquire severe COVID-19 infections and have a higher mortality rate [4]. Furthermore, it has been suggested that hypertension can lead to end-organ damage through physiological changes in the cardiac system such as fibrotic changes in heart muscles and hypertrophy of the left ventricle. This may increase the vulnerability of hypertensive patients’ hearts to COVID-19 [5].

In 2020, Wrapp et al. proposed that COVID-19 led to lung damage and failure as a result of its binding to angiotensin-converting enzyme 2 (ACE2) in pulmonary alveoli via their superficial spike proteins and consequently, lower levels of ACE2 will be obtained [6]. Thus, decreased amounts of ACE2 may result in elevated angiotensin II levels [1–7]. As angiotensin II is considered an essential factor in the renin–angiotensin–aldosterone system (RAAS) function. Elevated levels of angiotensin II can lead to vasoconstriction, sodium retention, free radical damage, inflammation, and fibrosis, which are known by their role to develop hypertension [7, 8]. The above studies were done at different times during the pandemic and on different sample sizes, thus resulting in a diversity of outcomes in hypertensive patients.

Herein, we present a systematic review and meta-analysis of the available literature to analyze the mortality rate of COVID-19 in hypertensive patients. Our aim is to provide the readers with the necessary information to predict the potential outcomes of the disease and assist in developing management plans.

## Methods

This meta-analysis was performed following the Preferred Reporting Items for Systematic Reviews and Meta-analysis (PRISMA) guidelines [9].

### Search Strategy

A comprehensive search was performed in PubMed, Cochrane, and Scopus databases published up to Jan 1st, 2019, and Feb 1st, 2022 using the terms COVID-19 OR SARS-CoV-2, Mortality OR Death AND Hypertension OR Hypertensive patients. We focused on published prospective, retrospective, cross-sectional, case–control, and cross-sectional keywords used for the Medical Subject Heading [MeSH] search included: (“SARS-CoV-2” [MeSH]) or “COVID-19” [MeSH]) or “coronavirus disease 2019” [MeSH]) “nCoV-2019” [MeSH]) or “coronavirus”” [MeSH]) and “Hypertension” [MeSH]) or “HTN” [MeSH]) and “Mortality” [MeSH]. Research, and preprints papers, that have been accepted for publication were also taken into consideration because there is currently a lack of evidence.

### Eligibility Criteria and Study Selection

The following criteria were used to determine which studies will be included in this review; (a) adult patients greater than 18 years; (b) hypertension as a comorbidity; (c) who was proven infected with the COVID-19 virus; (d) studies reporting mortality rates in these patients either prospective, retrospective, case–control or cross-sectional. The criteria included the above with the main interest in mortality in patients with hypertension, (e) English report publication. These were the exclusion criteria: (a) duplicate reports (including same patients’ information); (b) insufficient data; (c) reviews, and reports.

Three authors AA, DMS, and HA independently evaluated complete texts of articles and filtered them by the inclusion criteria. Where there were disagreements, discussions were made with senior authors ARA and RMZ until a consensus was established.

### Data Extraction

Three authors extracted variables from the information provided (first name of the author, study design, publication year, sample size, country, age, sex distribution, etc.) and according to the main stratification variable, author, country, data source, mean age, age range, study timeframe, baseline population group, total sample, mortality rate, and others.

### Statistical Analysis

Statistical analysis was done using STATA software version 17 and used the odds ratio (OR) or with a 95% confidence interval (CI) to estimate the correlation between mortality in patients with COVID-19 and hypertension. Cochran’s *Q* test was used to determine whether there was heterogeneity in effect sizes; a significant *Q* value suggests that there is heterogeneity rather than homogeneity. Using the *I*^2^ statistic, it was assessed what percentage of the overall variation may be attributed to study heterogeneity [10]. Funnel plots with the Egger regression test were used for assessing publication bias [11]. To graphically display the effect estimates from the included research, we used forest plots. Statistical significance was defined as *p* 0.05 at both ends.

### Ethics

The protocol for this systematic review was registered in the International Prospective Register of Systematic Reviews (PROSPERO) with unique No. of CRD42022358448.

## Result

### Study Selection

**Fig. 1:**
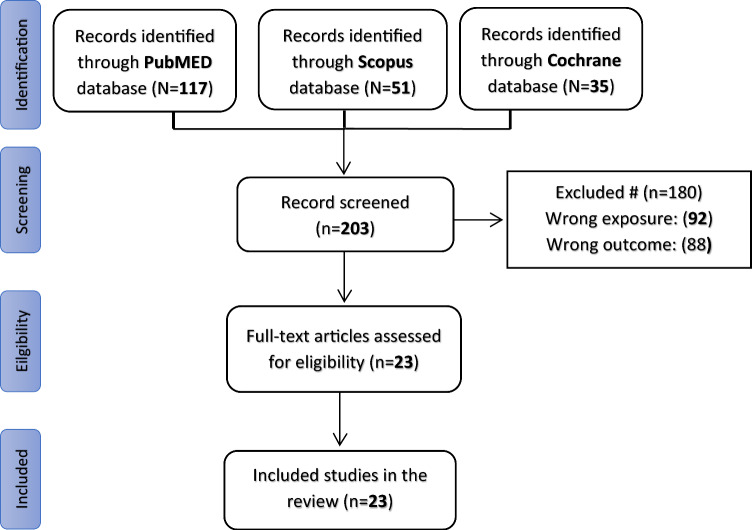
Flow chart of included studies

There were 203 studies found in the literature review. Out of them, 180 were excluded due to inclusion criteria not being met (wrong exposure, and wrong outcome). The remaining 23 were incorporated into our research study (Fig. 1).

### Assessment of Risk of Bias

Included studies were evaluated by the authors for external and internal validity using the bias assessment in incidence and prevalence studies [12]. The assessment of the risk of bias was conducted for all 23 papers included in the quantitative analysis. The result is reported in Table 1. From the 23 studies, 1 was found of moderate risk of bias, and the remaining 22 were found of low risk of bias.

**Table 1:** Assessment of bias per Hoy criteria described

Author, years	1	2	3	4	5	6	7	8	9	10	Score	Risk of bias
Huang et al. (2020) [13]	Y	Y	Y	Y	N	Y	Y	Y	Y	Y	9	Low
Yan et al. (2020) [14]	Y	N	Y	Y	N	Y	Y	Y	Y	Y	8	Low
Yuan et al. (2020) [15]	Y	Y	Y	Y	N	Y	Y	N	Y	Y	8	Low
Fu et al. (2020) [16]	Y	Y	Y	Y	N	Y	Y	Y	N	Y	8	Low
Sun et al. (2020) [17]	Y	Y	Y	Y	N	Y	Y	N	Y	Y	8	Low
Hui et al. (2020) [18]	Y	Y	Y	Y	N	Y	Y	N	Y	Y	8	Low
Shi et al. (2020) [19]	Y	Y	Y	Y	N	Y	Y	N	Y	Y	8	Low
Klang et al. (2020) [20]	Y	Y	Y	Y	N	Y	Y	N	Y	Y	8	Low
Gao et al. (2020) [21]	Y	Y	Y	Y	N	Y	Y	N	Y	Y	8	Low
Marjot et al. (2022) [22]	Y	Y	Y	Y	N	Y	Y	Y	N	Y	8	Low
Joy et al. (2020) [23]	Y	Y	Y	Y	N	Y	Y	Y	Y	Y	9	Low
Kompaniyets et al. (2021) [24]	N	Y	Y	Y	N	N	Y	N	Y	Y	6	Moderate
Zhou et al. (2020) [25]	Y	Y	Y	Y	N	Y	Y	N	Y	Y	8	Low
Meng et al. (2020) [26]	Y	Y	Y	Y	N	Y	Y	Y	N	Y	8	Low
Caizheng et al. (2020) [27]	Y	Y	Y	Y	N	Y	Y	Y	N	Y	8	Low
Wang et al. (2020) [28]	Y	Y	Y	Y	N	Y	Y	N	Y	Y	8	Low
Chilimuri et al. (2020) [29]	Y	Y	Y	Y	N	Y	Y	N	Y	Y	8	Low
Lee et al. (2020) [30]	Y	N	Y	Y	N	Y	Y	Y	N	Y	7	Low
Gu et al. (2020) [31]	Y	Y	Y	Y	N	Y	Y	Y	Y	Y	9	Low
Atkins (2020) [32]	Y	Y	Y	Y	N	Y	Y	N	Y	Y	8	Low
Du et al. (2020) [33]	Y	Y	Y	Y	N	Y	Y	Y	Y	Y	9	Low
Bhatia et al. (2021) [34]	Y	Y	Y	Y	N	Y	Y	N	Y	Y	8	Low
Xu et al. (2020) [35]	Y	Y	Y	Y	N	Y	Y	N	Y	Y	8	Low

Risk of bias item	Risk of bias levels	Risk of bias result
1. Was the study’s target population a close representation of the national population about relevant variables, e.g., age, sex, occupation? 2. Was the sampling frame a true or close representation of the target population? 3. Was some form of random selection used to select the sample, OR, was a census undertaken? 4. Was the likelihood of non-response bias minimal? 5. Were data collected directly from the subjects (as opposed to a proxy)? 6. Was an acceptable case definition used in the study? 7. Was the study instrument that measured the parameter of interest shown to have reliability and validity? 8. Was the same mode of data collection used for all subjects? 9. Were the numerator(s) and denominator(s) for the parameter of interest appropriate 10. Summary of the overall risk of study bias	Yes = low risk = 0 No = high risk = 1	Low risk = 0–3 Moderate risk = 4–6 High risk = 7–9

### The study Included Characteristics

A total of 23 studies (18 were retrospective, 2 were observational, 1 was cross-section, 1 was a nested case–control, and 1 was prospective) involving 6,11,522 patients from 4 countries (14 from china, 1 from Korea, 3 from the UK, 1 from Australia, and 4 from the USA) were included in our study. The studies were published from 2020 to 2021(Table 2). The confirmed number of COVID-19 with HTN cases in each study ranged from 5 to 9964. The mortality ranged from 0.17% to 31% in different studies.

**Table 2:** Characteristics of the included studies

Author, year	Country	Study design	Patients no.	Age (mean)	Enrollment period
Atkins et al. (2020) [32]	UK	Retrospective	507	74.7 (4.4)	(Mar–Apr 2020)
Caizheng et al. (2020) [27]	China	Retrospective	1464	64.0 (51‒71)	(Jan–Feb 2020)
Chilimuri et al. (2020) [29]	USA	Retrospective	375	63.0 (52–72)	(Mar–Apr 2020)
Du et al. (2020) [33]	China	Prospective	179	57.6 ± 13.7	(Dec 2019–Feb 2020)
Fu et al. (2020) [16]	China	Retrospective	200	N/A	Jan–Jan 2020
Gao et al. (2020) [21]	China	Retrospective	2877	55.38–64.26	Feb–Mar 2020
Gu et al. (2020) [31]	China	Nested case–control	275	72.5	Dec 2019–March 2020
Huang et al. (2020) [13]	China	Retrospective	310	62 (40–70)	2020
Joy et al. (2020) [23]	UK	Observational	56,628	> 64 (65–74) > 75	Jan–May 2019, May 2020
Klang et al. (2020) [20]	USA	Retrospective	3406	68.0 (60.0–77.0)	Mar–May 2020
Wang et al. (2020) [28]	CHINA	Retrospective	296	47.32 ± 14.95	Jan–Feb 2020
Lee et al. (2020) [30]	Korea	Retrospective	98	71.0 (67.0–78.0)	Feb–Mar 2020
Meng et al. (2020) [26]	China	Retrospective	109	> 18	Jan–Mar 2020
Shi et al. (2020) [19]	China	Retrospective	671	63 (50–72)	Jan–Feb 2020
Sun et al. (2020) [17]	China	Retrospective	244	> = 60 (64–78)	Jan–Mar 2020
Xu et al. (2020) [35]	China	Retrospective	703	60.5 ± 17.2	Jan–Mar 2020
Yan et al. (2020) [14]	China	Retrospective	1004	68	Jan–Mar 2020
Yuan et al. (2020) [15]	China	Retrospective	27	60 (47–69)	Jan–Jan 2020
Zhou et al. (2020) [25]	China	Retrospective	191	56·0 (46–67)	Dec 2019–Jan 2020
Kompaniyets et al. (2021) [24]	USA	Cross-sectional	540,612	66 (53–77	Mar 2020–Mar 2021
Bhatia et al. (2021) [34]	Australia	Observational	546	62.9 ± 19.8	NA
Marjot et al. (2022) [22]	UK	Retrospective	745	59 (47–68)	Mar–Jul 2020
Total			611,522		

### Characteristics of the Included Patients

The characteristics of the studies included in the analysis are summarized in Table 2. A total of 6,11,522 patients were included, with males representing 50.7% of the study population, resulting is a male-to-female ratio of approximately 1.1:1. The age range of the patients was 33–81 years. Of the total 23 studies that included severity as part of the composite endpoint, the reasons were defined as follows: pre-defined criteria (11 studies); ICU requirement (7 studies); ventilation requirement (4 studies); ARDS (1 study).

### Hypertension with COVID-19 Mortality

**Fig. 2:**
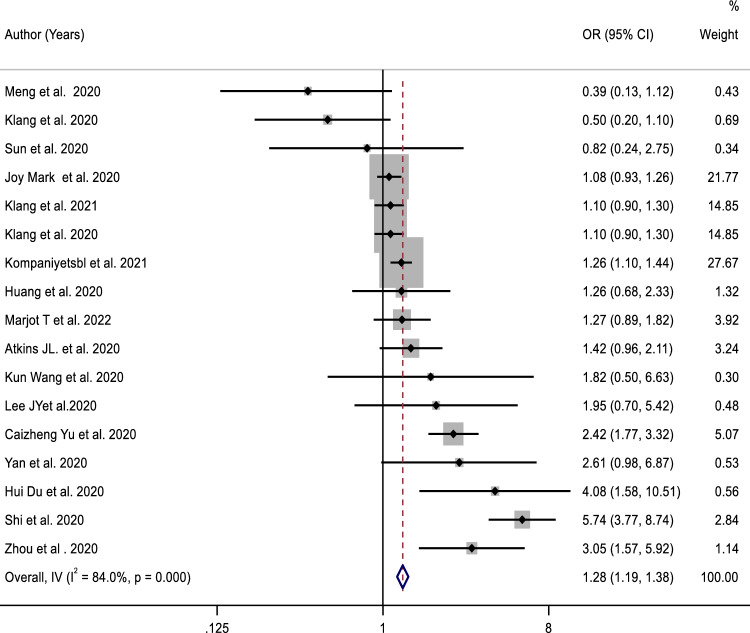
Forest plot for the association between hypertension and mortality in patients with COVID-19

Patients who had both COVID-19 and hypertension were found to have an increased risk of mortality to those who only had COVID-19. The odds ratio (OR) was 1.28 with a 95% confidence interval (CI) of 1.20–1.39. There were higher heterogeneity in the studies, with a Q value of 105.93 *p* < 0.001, and *I*-squared (*I*^2^) value of 84% (Fig. 2). From this plot, the mortality rate in the included studies ranged from a minimum of 0.39 (95% CI 0.13–1.12) [26] to a maximum of 5.74 (95% CI 3.77–8.74) [36]. Out of the total of 6,11,522 patients, 3119 died, resulting in an overall mortality prevalence of 0.5%.

The results of subgroup analyses suggest that male patients with hypertension with COVID-19 had a slightly lower risk of mortality than female patients. This was observed in patients where the percentage of men was greater than 50% (OR 1.33; 95% CI 1.01–1.76) and in patients where the percentage of men was less than or equal to 50% (OR 2.26; 95% CI 1.15–4.48) (Table 3). In terms of geography, China had a significantly higher mortality rate [OR 2.07; 95% CI (1.22, 3.50)] compared to other countries [OR 1.52; 95% CI (1.07, 1.25)]. Meta-regression analysis also showed a significant impact of countries on the association between hypertension and mortality in COVID-19 patients (β − 0.265; *p* = 0:018). However, studies with a sample size of ≤ 1000 reported a stronger association between hypertension and COVID-19 mortality [OR 1.78; 95% CI (1.10, 2.87)] compared to studies with a sample size of > 1000 [OR 1.26; 95% CI (0.95, 1.69)].

**Table 3:** Subgroup analysis of the association between critical COVID-19 and hypertension and mortality

Subgroups	Study (*n*)	OR (95%CI)	Heterogeneity test		
			*Q*	*p* value	*I*^2^ (%)
Overall	17	1.28 (1.19, 1.38)	105.93	< 0.001	84.0%
Geography					
China	9	2.07 (1.22, 3.50)	35.87	< 0.001	80.87
Non-China	8	1.52 (1.07, 1.25)	8.93	0.258	4.1
The percentage of men					
≤ 50%	5	2.26 (1.146, 4.48)	56.27	< 0.001	86.1
> 50%	12	1.33 (1.01, 1.76)	43.37	< 0.001	86.7
Sample size					
≤ 1000	10	1.78 (1.10, 2.87)	49.83	< 0.001	81.4
> 1000	7	1.26 (0.95, 1.69)	29.67	< 0.001	90.7

### Publication Bias

**Fig. 3:**
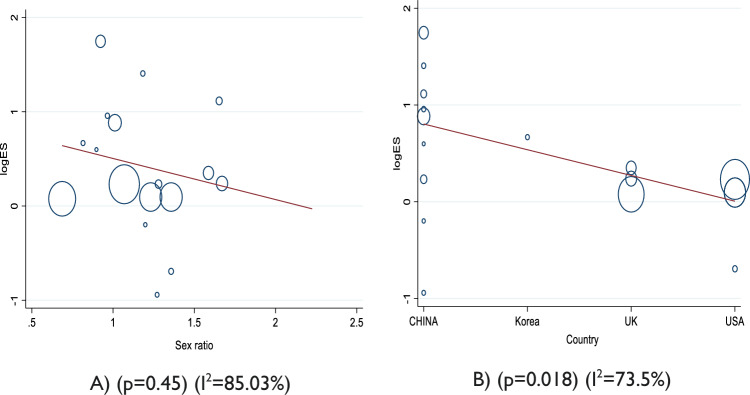
Random-effects meta-regression analysis of the effect of sex and countries on the association between hypertension with COVID-19 and mortality (**A**, **B**)

No publication bias was detected in the current meta-analysis, although slight asymmetries were observed in the funnel plots (Fig. 3); we used Egger’s linear regression test to see the publication bias β − 0.02; (*p* = 0.980) which is not statistically significant.

## Discussion

Since the emergence of COVID-19 as a pandemic, there has been an increased prevalence of cases, which has led to a rise in mortality rates among patients with non-communicable disease. During the COVID-19 pandemic, many countries have recommended that people stay at home, and hospital services have also been limited with a primary focus on COVID-19 patients and emergency cases to limit exposure to the virus and its spread. As a result, there has been a decrease in care for non-communicable disease patients who have been diagnosed with hypertension. This change in level of care may lead to an increase in the mortality rate among these patients.

Studies have indicated that COVID-19 infections have a greater impact on older males with comorbidities, leading to increased mortality rates in this group [37, 38]. It is interesting to note that MERS and SARS infections have been reported to be more prevalence in males than females [39–41]. This may be due to differences in the types and levels of sex hormones between the genders, which could increase the susceptibility to COVID-19 infection. However, our analysis revealed that male patients had a slightly lower risk of mortality compared to female patients. Several studies have shown that individuals with high blood pressure are at a higher risk of severe infection and death from COVID-19 than healthy individuals [13, 40, 42, 43]. In addition, based on the literature, there are other risk factors that increase COVID-19 mortality, such as patients who have been diagnosed with more than one non-communicable disease; including such as diabetes, CVDs, respiratory disease, cancer, and others [44].

The study by Kampaniyets et al. found that younger patients (aged 18–39) had a higher risk of severe COVID-19 cases, while older patients had a lower risk [24]. Similarly, Klang et al. found that younger patients with a BMI ≥ 40 were associated with higher mortality rates [20]. The study by Gao et al. compared hypertensive patients who took antihypertensive medications to those who did not. The study found that those who did not take antihypertensive medications had an increased risk of mortality, possibly due to their ACE2 levels. In addition, the study found that there was no statistically significant difference in mortality rate, COVID-19 severity, and ventilation requirements between hypertensive patients treated with RAAS inhibitors and those treated with non-RAAS inhibitors [21]. However, Sun et al. suggested that antihypertensive drugs like ACE2 inhibitors should be used with caution in patients infected with COVID-19 [17].

### Strength and Limitation

To our knowledge, this is the first systematic review and meta-analysis that quantifies the effect of hypertension on COVID-19 mortality. Also, we reviewed the hypertension prevalence among COVID-19 patients with a large sample size. We employed a rigorous review procedure and followed PRISMA principles. We employed a rigorous approach to identifying papers, extracting data, and appraising data after searching different electronic databases. We also established operational definitions for the results, separating them using simple and repeatable formulas. We also avoided using duplicate publications, which could have skewed the interpretation of the prevalence incidence values and to reduce the effect of multiple-publication bias in the study. This review has some restrictions as well. We eliminated non-English papers due to a lack of resources. Moreover, substantial heterogeneity makes a debate about whether pooling prevalence is a worthy procedure to do.

## Conclusion

In conclusion, the meta-analysis suggests that hypertensive patients who get infected with COVID-19 have a significantly higher death probability compared to those without hypertension. However, hypertension may not be the only risk factor, and as a combination of other comorbidities and age also seem to increase the risk of mortality. The findings of the current systematic review and meta-analysis could aid hospitals in identifying high-risk patients and to reassessing their priorities for managing chronic diseases and minimize the adverse effects of future pandemics.

## Data Availability

All data analyzed during this study are included in this article, and further inquiries can be directed to the corresponding author.

## Abbreviations

COVID-19: Coronavirus disease of 2019
PRISMA: Preferred Reporting Items for Systematic Reviews and Meta-analyses
ACE2: Angiotensin-converting enzyme 2
RAAS: Renin–angiotensin–aldosterone system
